# Special Issue “Sustainable Nutrition—Healthy People”

**DOI:** 10.3390/nu15143199

**Published:** 2023-07-19

**Authors:** Emilia Vassilopoulou

**Affiliations:** 1 Pediatric Unit, Fondazione IRCCS Ca’ Granda Ospedale Maggiore Policlinico, 20122 Milan, Italy; vassilopoulouemilia@gmail.com; 2 Department of Nutritional Sciences and Dietetics, International Hellenic University, 57400 Thessaloniki, Greece

Food security is defined as the situation in which all people have access to their preferred food, which is simultaneously sufficient in quantity, safe, nutritious, and covers their dietary needs in order for them to remain active and healthy [1]. This multidimensional definition states that for food security to be achieved, food must be available in sufficient quantities and stably accessible to avoid nutritional deficiencies and consumed, at all times, without the risk of non-food contamination. The necessary conditions to ensure are the population’s (a) psychosomatic well-being, (b) nutritional education on how to make proper food choices, and (c) access to food processed safely.

It is only a few decades ago that the phenomenon of globalization started to manifest itself in major changes in lifestyle and diet, but these changes evolved at such a rapid pace that the human race did not have time to adapt properly. Food insecurity arose sharply in less than a decade, under the pressure of the economic crisis, the rapid progression of climate change, the consequences of the COVID-19 pandemic, high energy prices, and other factors [2]. Thereafter, the phenomenon of the “triple burden of malnutrition” occurred which encapsulates the coexistence of (a) malnutrition due to poverty; (b) micronutrient deficiencies as a result of the food supply chain producing and marketing cheap, low-quality foods; and (c) overnutrition due to poor dietary choices resulting from a lack of knowledge on which foods are nourishing and which should be preferred for a healthy diet [3].

In the Western world, a large proportion of the population has moved from rural settings to semi-urban or urban environments, with this having dramatic effects on the lifestyle of a large number of people. Traditional eating models have been replaced by patterns in which an individual’s diet is characterized by the over-consumption of ultra-processed foods (UPFs), which are high in sugar, fat, and salt, and they are often accompanied by the unsuitable use of dietary supplements [4]. These dietary changes are associated with increased rates of obesity in all age groups [5], even in critical periods such as pregnancy [6]. In parallel, there is documented evidence of the earlier onset of non-communicable diseases, including cardiovascular disease, type II diabetes mellitus, and cancer, and an increased prevalence of allergies [7].

Various conditions such as irritable bowel disease have been shown to be correlated with lower adherence to traditional dietary patterns such as the Mediterranean diet and low levels of vitamin D, which are also related to the reduction in hours spent outside in the urban environment [8].

Young children and adults may strive to follow “healthy diet models”, but because of their limited knowledge of how to put the remarkable amounts of information they receive via the mass media into practice, they risk falling into the trap of developing eating disorders, such as orthorexia [9].

The wide range of studies included in this Special Issue highlights the need for the active promotion of more sustainable diets that: (a) respect the environment, (b) meet the nutritional needs of diverse populations, (c) reduce the issues brought about by the inefficient and inappropriate distribution of food, and (d) facilitate the management of the dual phenomena of obesity and malnutrition, apparent to varying degrees in different countries around the world [10,11,12,13,14].

For an effective transition to healthier and more sustainable diets, an in-depth understanding of current dietary patterns and their associated environmental impact is required [11,14,15,16].

The meaning of a sustainable diet should account not only for survival and balance with our environment but also for finding a holistic symmetry for ourselves in the new environment, which we have created but to which we were not prepared to adapt, and to live healthily in terms of both somatic and psychological health.
